# Autologous Protein Solution processing alters lymphoid and myeloid cell populations and modulates gene expression dependent on cell type

**DOI:** 10.1186/s13075-022-02875-x

**Published:** 2022-09-12

**Authors:** Alexis N. Peña, Sven D. Sommerfeld, Amy E. Anderson, Jin Han, David R. Maestas, Joscelyn C. Mejias, Jennifer Woodell-May, William King, Sudipto Ganguly, Jennifer H. Elisseeff

**Affiliations:** 1 Translational Tissue Engineering Center, Johns Hopkins University, 400 N. Broadway Smith Building 5th floor, Baltimore, MD 21231 USA; 2 Department of Biomedical Engineering, Johns Hopkins University, Baltimore, MD USA; 3 Wilmer Eye Institute, Johns Hopkins University, Baltimore, MD USA; 4 Department of Cellular and Molecular Medicine, Johns Hopkins School of Medicine, Baltimore, MD USA; 5 Zimmer Biomet, 56 East Bell Drive, Warsaw, IN 46581 USA; 6 Bloomberg~Kimmel Institute for Cancer Immunotherapy and Sidney Kimmel Comprehensive Cancer Center, Johns Hopkins University, School of Medicine, Baltimore, MD USA; 7 Department of Oncology and Sidney Kimmel Comprehensive Cancer Center, Johns Hopkins University School of Medicine, Baltimore, MD USA

**Keywords:** Blood derivative, Platelets, Growth factors, Autologous cell-based therapies, Knee osteoarthritis, Cytokines, Injection

## Abstract

Osteoarthritis (OA) is a degenerative disease associated with cartilage degradation, osteophyte formation, and fibrillation. Autologous Protein Solution (APS), a type of autologous anti-inflammatory orthobiologic, is used for pain management and treatment of OA. Various compositions of autologous PRP formulations are in clinical use for musculoskeletal pathologies, by nature of their minimal processing and source of bioactive molecules. Currently, there is no consensus on the optimal composition of the complex mixture. In this study, we focused on elucidating the immune cell subtypes and phenotypes in APS. We identified the immune cell types in APS from healthy donors and investigated phenotypic changes in the immune cells after APS processing. Based on flow cytometric analysis, we found that neutrophils and T cells are the most abundant immune cell types in APS, while monocytes experience the largest fold change in concentration compared to WBCs. Gene expression profiling revealed that APS processing results in differential gene expression changes dependent on immune cell type, with the most significantly differentially regulated genes occurring in the monocytes. Our results demonstrate that the mechanical processing of blood, whose main purpose is enrichment and separation, can alter its protein and cellular composition, as well as cellular phenotypes in the final product.

**Supplementary Information:**

The online version contains supplementary material available at 10.1186/s13075-022-02875-x.

## Background

Blood-derived therapies provide high concentrations of autologous mitogenic and chemotactic cytokines and growth factors. Autologous platelet-rich plasma (PRP) therapy, a blood-derived therapy or autologous orthobiologic, is comprised in large part of concentrated platelets and has widespread clinical use in chronic pathologies of the musculoskeletal system. PRP contains varying concentrations of platelets, leukocytes, red blood cells, clotting factors, growth factors, bioactive proteins, RNAs, and lipids. PRP contains more than 1500 bioactive factors such as platelet-derived growth factor-BB, transforming growth factor-β1, vascular endothelial growth factor, basic fibroblast factor, endothelial growth factor, and insulin-like growth factor-1 [1]. PRP’s main mode of action is purported to be due to the supraphysiologic concentrations of growth factors and hormones released upon platelet activation from secretory granules, the most abundant being α-granules, that can change the catabolic environment and subsequently modulate reparative and regenerative processes locally [1–4]. The role of the non-platelet cells in the context of PRP therapy has yet to be fully understood. The known functions of non-platelet cells in wound healing could play a role in PRP’s mode of action. The wound healing cascade is tightly regulated and requires coordination among all components of blood, with non-platelet cells like granulocytes and monocytes playing a role in releasing factors, cell recruitment, and promoting cellular phenotypic changes.

Knee osteoarthritis (OA), a degenerative multifactorial disease, is the most common form of arthritis. Knee OA is characterized by joint pain and structural changes including stiffness, synovial inflammation and fibrosis, cartilage erosion, subchondral bone alterations, osteophyte formation, and can lead to loss of motion in the joint. The chronic low-grade inflammatory and catabolic microenvironment in the diseased joint contributes to OA progression and is driven by cytokines, chemokines, and proteolytic enzymes. The pro-inflammatory cytokines, interleukin-1 beta (IL-1β), and tumor necrosis factor alpha (TNF-α) can induce catabolism in the joint by increasing nitric oxide and reactive oxygen species (ROS) in fibroblast-like synoviocytes (FLS). IL-1β and TNF-α can promote the production of ECM-degrading enzymes and inhibitors which can lead to further cartilage destruction and OA disease progression [5, 6]. Knee OA is a complex disease comprising multiple interactions and crosstalk between supportive and effector cell types such as endothelial cells, FLS, macrophages, and T cells. This complexity requires a multifunctional therapy that can act on the various modes of disease progression such as immune modulation, inhibiting inflammation, and chondrocyte/fibroblast reprogramming. Although there are treatment options that span from non-pharmacologic approaches to surgical procedures; pain management and treatment remain areas of significant clinical unmet need. Preclinical and clinical studies support the use of orthobiologics including PRP therapy for the treatment of knee OA [7]. Orthobiologics like PRP are minimally invasive approaches with limited costs. Systematic reviews of clinical evidence suggest that intra-articular injections of PRP with low-WBCs in patients with knee OA reduce pain based on multiple validated outcome scores (Western Ontario and McMaster Universities Osteoarthritis Index or Lequesne index; Knee Injury and Osteoarthritis Outcome Score scale, OMERACT-OARSI) [2, 8]. Meta-analysis of randomized control trials (RCTs) evaluating PRP broadly for knee OA compared to saline or other intra-articular treatments such as hyaluronic acid and steroid injections found that PRP injections provide better clinical outcomes than other injectables, and its clinical benefits increase over time [9].

Autologous therapies can be prepared from the whole blood using different processing techniques that result in different compositions of PRP. PRP can be classified as pure PRP, leukocyte-poor PRP, or leukocyte-rich PRP, although a standard classification system does not exist [8]. Patient variability and different processing techniques can cause varying levels of platelets, platelet activation rates, and growth factor profiles in PRP [1]. Additionally, patient variability can arise from differences in existing comorbidities, gender, age, and lifestyle. These factors may impact the functional capacity of the PRP therapy. Time of blood acquisition may also impact PRP composition due to the circadian variation of platelet concentration, function, and concentration of bioactive factors [3, 10]. To date, there are no therapeutically relevant guidelines on the composition of PRP or the number of injections required for OA treatment. There is no consensus and limited clinical evidence for the use of PRP containing WBCs for musculoskeletal pathologies. Some studies have found that the addition of leukocytes contributes to transient inflammation and patient discomfort that, in part, could be due to the damaging effects of proteases and reactive oxygen species (ROS) released by granulocytes [11, 12]. Other studies have suggested that leukocytes may not contribute to the anabolic environment required for disease treatment [13]. Conversely, others have noted that WBCs are the source of important cytokines and enzymes needed in wound healing [3]. Further clinical evaluation is needed to understand the contribution of WBCs to PRP therapy.

nSTRIDE® Autologous Protein Solution (APS), also referred to as Pro-Stride APS in animal health, is an autologous therapy for pain management and treatment of OA. Preclinical studies and characterization of APS have demonstrated its ability to modulate the inflammatory response and reduce cartilage degradation [7]. APS is composed of WBCs, platelets, and concentrated plasma. Previous characterization of APS has shown an enrichment of anti-inflammatory cytokines and anabolic growth factors such as IL-1Ra, sIL-1RII, and sTNF-R1 compared to whole blood [4]. To date, a clinical trial with a single injection of APS for the treatment of OA has demonstrated significant improvement in pain when compared to baseline and APS’ safety profile is comparable to those that were in the saline-treated groups [14, 15]. In addition to the therapeutic importance of anti-inflammatory cytokines and anabolic growth factors present in PRP therapies broadly, in vitro studies have found that biomolecules are more concentrated when leukocytes are present in an orthobiologic [16]. Differences in platelet degranulation based on PRP composition suggest that degranulation processes are complex and have multiple factors that can influence the extent of degranulation. Leukocytes may provide important intracellular factors such as IL-1Ra, influence the degranulation of bioactive molecules, produce chemokines/cytokines, and can regulate the immune response. These results have warranted further investigation into the WBC composition and cell phenotypes in APS.

In the present study, we investigated the immune cell and protein composition in APS and explored how processing may impact cell phenotypes and gene expression in healthy donors before injections. We found that APS processing enriched CD45^+^ immune cells, with neutrophils and T cells being the most abundant immune cell types present in the final product. Monocytes experienced the highest fold change in enrichment when compared to WBCs. APS processing maintained the dominant anti-inflammatory CD163^+^ subpopulation among classical and non-classical monocytes that was found in WBCs. Immune subpopulations were sorted for gene expression profiling using a multiplex gene expression assay. Monocytes experienced significant gene expression changes due to APS processing with differential expression in genes related to antigen processing and presentation. An in vivo murine study using a post-traumatic osteoarthritis (PTOA) model was performed to evaluate the durability of APS immune cell components. The study found that the immune cells persisted and suggests that the non-platelet cells can provide durable cues and soluble factors beyond the initial injection.

## Results

### APS processing enriches immune concentrations of multiple immune cell populations

**Fig. 1:**
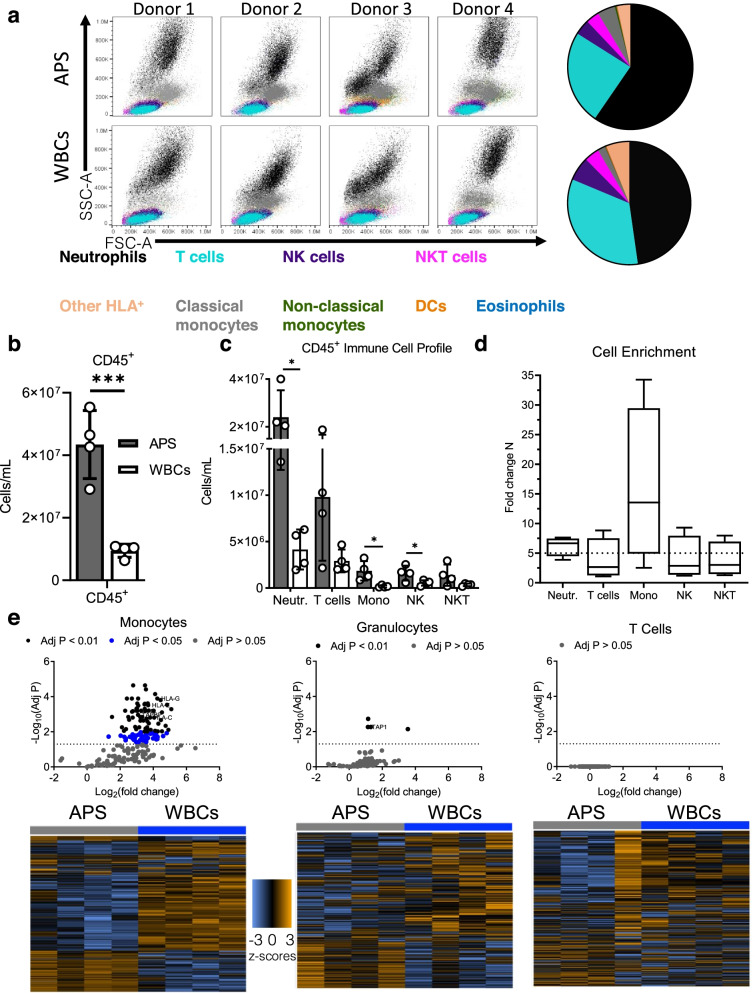
Enrichment and differential gene expression after APS processing is dependent on immune cell type. Blood from donors was either processed as WBCs or APS for flow cytometry. Monocytes, granulocytes, and T cells were sorted from WBCs and APS for multiplex gene expression analysis using the Nanostring platform. **a** Flow cytometry plots of APS and WBCs for all donors with pie charts representing the average defined immune cell types by the same colors shown in the flow plots, neutrophils (CD45^+^CD3^−^CD15^+^CD16^+^), T cells (CD45^+^CD3^+^CD14^−^CD16^−^), NK cells (CD45^+^CD3^-^CD15^−^CD14^-^CD16^+^HLA^−^), NKT cells (CD45^+^CD3^+^CD14^−^CD16^+^), other HLA^+^ (CD45^+^CD3^−^CD15^−^CD14^−^HLA^+^CD16^−^CD11c^−^), classical monocytes (CD45^+^CD3^−^CD15^−^CD14^+^CD16^dim^), non-classical monocytes (CD45^+^CD3^−^CD15^−^CD14^dim^CD16^+^), DCs (CD45^+^CD3^−^CD15^−^CD14^−^HLA^+^CD16^−^CD11c^+^HLA^+^), and eosinophils (CD45^+^CD3^−^CD15^+^CD16^−^). **b** Flow cytometric analysis of CD45^+^ cell concentration from donors (*n* = 4) in APS and WBCs (mean with ± SD). **c** Flow cytometric analysis of CD45^+^ immune cell subpopulation concentrations, Neutrophils (Neutr.), T cells, monocytes (Mono), NK cells (NK), and NKT cells (NKT) (mean with ± SD). **d** Quantification of the fold change of the immune subpopulations in APS compared to WBCs, (whiskers represent min to max), the line at *Y* = 5. **e** Volcano plots of differentially regulated genes in the sorted immune cell populations (monocytes, granulocytes, and T cells) from APS compared to WBCs. The significance of differential gene regulation was determined by calculating *p*-value adjustment using the Benjamini-Hochberg method of estimating false discovery rates (FDR), (*p*_adj_ < 0.05, 1.5 ≤ LFC ≤ − 1.5). Heat map of sorted monocytes, granulocytes, and T cells from APS and WBCs normalized *z*-scores, scaled to give all genes equal variance, generated via unsupervised clustering. Multiple unpaired *t*-test without correction for multiple comparisons, alpha = 0.05 for **b** and **c**). *p < 0.05, ***p < 0.001

**Fig. 2:**
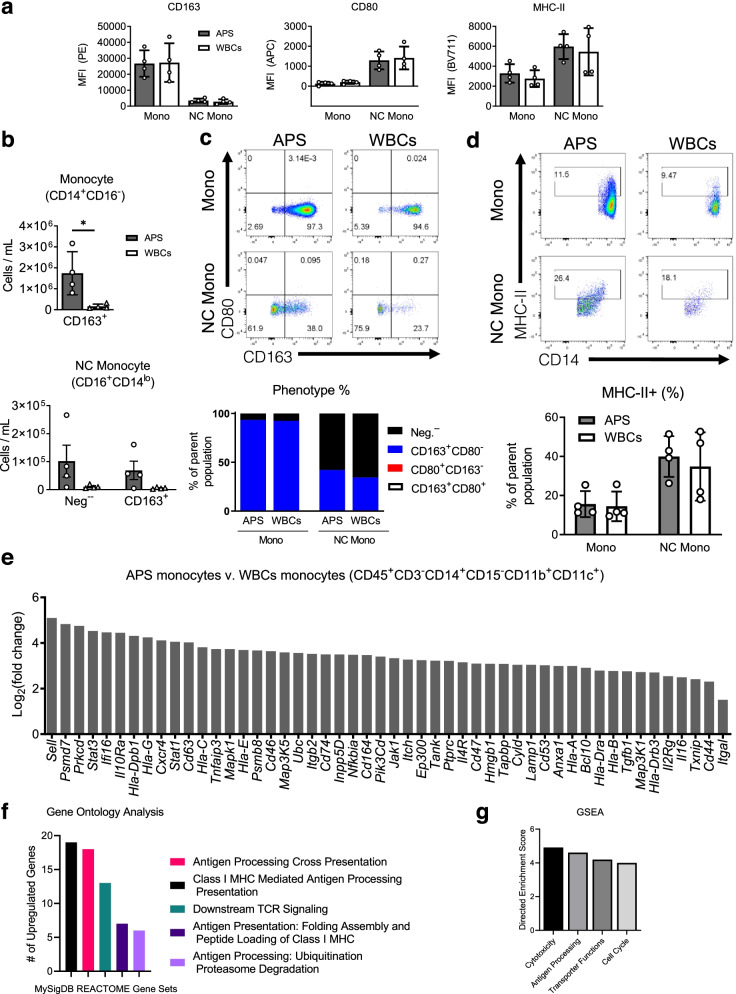
Monocytes maintain M2-like polarization and experience significant changes in the gene expression after APS processing. **a** Mean fluorescence intensity (MFI) of hallmark macrophage polarization markers CD80 (M1) and CD163 (M2) and MHC-II in classical monocytes (CD45^+^CD^−^CD15^−^CD14^+^CD16^dim^) and non-classical monocytes (CD45^+^CD3^−^CD15^−^CD14^dim^CD16^+^). **b** Flow cytometry results of CD163^+^ cell concentration in classical monocytes and CD163^+^ and double negative (Neg^−^) in non-classical monocyte in APS and WBCs. Individual donors are denoted as circles for APS and triangles for WBCs. **c** Flow cytometry plots of classical and non-classical monocytes subtyping based on CD80 and CD163 in APS and WBCs. Phenotype plots of the percentage of CD80^+^, CD163^+^, CD80^+^CD163^+^, and double negative (Neg^−^) in classical and non-classical monocytes. **d** Flow cytometry plots of MHC-II^+^ classical monocytes and non-classical monocytes and quantification of the percentage of MHC-II^+^ in classical and non-classical monocytes in APS and WBCs, data are mean with ± SD. **e** Differential expression of the top 50 significantly upregulated genes in APS-sorted monocytes compared to WBCs-sorted monocytes (CD45^+^CD3^−^CD14^+^CD15^−^CD11c^+^CD11b^+^) from Nanostring gene expression assay. **f** Gene Ontology analysis of Nanostring gene expression data using MySigDB REACTOME Gene sets, *p*_adj_ < 0.05. **g** Gene set enrichment analysis (GSEA) using Nanostring Annotations v46. For all bar graphs, data are mean with ± SD. Multiple unpaired *t*-test without correction for multiple comparisons with set *p*-value threshold, alpha = 0.05 for **b**. *p < 0.05

We characterized the immune cell populations after APS processing in blood acquired from 4 healthy human donors, Fig. 1a. We used flow cytometry to investigate the immune cell types present in APS; we defined populations of neutrophils (CD45^+^CD3^−^CD15^+^CD16^+^), monocytes (CD45^+^CD3^−^CD15^−^CD14^+^CD16^dim^), natural killer cells (NK) cells (CD45^+^CD3^−^CD15^−^CD14^−^CD16^+^HLA^−^), NKT cells (CD45^+^CD3^+^CD14^−^CD16^+^), and T cells (CD45^+^CD3^+^CD14^−^CD16^−^) based on cell surface markers, gating strategy shown in Fig. S1a. APS processing resulted in a significant 5-fold enrichment of CD45^+^ immune cells while maintaining cell viability when compared to WBCs (Fig. 1b). Neutrophils (24 million ± 11 million cells/mL) and T cells (9.8 million ± 6.9 million cells/mL) were the most abundant immune cell types in APS followed by classical monocytes (1.9 million ± 1 million cells/mL), NK cells (1.6 million ± 0.80 million cells/mL), and NKT cells (1.4 million ± 1.1 million cells/mL) (Fig. 2c). Neutrophils, classical monocytes, and NK cells were significantly enriched in APS compared to WBCs. Other immune cell types were enriched after processing and present at lower concentrations including non-classical monocytes (CD45^+^CD3^−^CD15^−^CD14^dim^CD16^+^), dendritic cells (CD45^+^CD3^−^CD15^−^CD14^-^HLA^+^CD16^−^CD11c^+^HLA^+^), and eosinophils (CD45^+^CD3^−^CD15^+^CD16^−^) (Fig. S2a-b). Eosinophils experienced significant enrichment after APS processing.

We then explored the donor variability. Donor samples were processed on the same day, and the flow cytometry experiment was performed on the same cytometer at the same settings. The variation among the same immune cell types between donors shown in the forward and side scatter plots suggests donor differences (Fig. 1a). There are apparent shifts in the subpopulations between individual donors. The classical monocytes (CD45^+^CD3^−^CD15^−^CD14^+^CD16^dim^, gray) are more abundant in APS relative to WBCs and shift among different donors in the forward and side scatter plots. The neutrophils (CD45^+^CD3^−^CD15^+^CD16^+^, black), in particular, have large variability between volunteers in terms of size and granularity. Shifts among granulocytes due to density changes during inflammation suggest differences in activation status, maturation state, and granulation [17, 18]. Overall, after APS processing, monocytes (16 times more abundant), followed by neutrophils (6 times more abundant), had the largest fold change among the immune cell populations when compared to WBCs (Fig. 1d).

### Distinct differential gene expression changes in immune cell subtypes after APS processing is immune cell type-dependent

We sorted granulocytes (CD45^+^CD11b^+^CD3^−^CD15^+^CD14^−^), T cells (CD45^+^CD3^+^CD11b), and monocytes (CD45^+^CD3^−^CD14^+^CD15^−^CD11b^+^CD11c^+^) from APS and WBCs to evaluate the mechanistic pathway activity (FACS gating strategy shown in Fig. S3). To assess the gene expression changes related to the innate and adaptive immune response, we performed the Nanostring multiplex gene expression assay using the PanCancer Immune Profiling CodeSet. Data were analyzed using the ROSALIND platform (*p*_adj_ < 0.05, 1.5 ≤ LFC ≤  − 1.5), and analysis revealed differential changes in APS compared to WBCs dependent on cell type. The ROSALIND platform is a cloud-based software platform for gene expression data analysis. With respect to the genes analyzed, T cells remained largely unaffected after APS processing with no significant differentially expressed genes when compared to T cells sorted from WBCs*.* The myeloid compartment experienced significant changes after APS processing; 109 and 4 genes were differentially regulated from sorted monocytes and granulocytes, respectively (Fig. 1e). The differentially regulated genes in sorted granulocytes were related to antigen processing (*Tap1*) and inflammatory responses (*Mapkapk2*, *Ptgs2*) (Fig. S2c). We used STRING to construct a protein-protein interaction (PPI) network of the differentially regulated genes. Using the KEGG Pathways database we found functional enrichment of the VEGF signaling pathway (*Mapkapk2, Ptgs2*) and c-type lectin receptor (CLR) signaling pathway (*Mapkapk2*, *Ptgs2*). VEGF signaling has an important role in leukocyte recruitment and is involved in the wound healing cascade by promoting endothelial cell proliferation and angiogenesis, while CLRs can control adaptive immunity. The differences in the gene expression, particularly those found in monocytes after APS processing, may suggest functional changes that could impact treatment outcomes. Notably, all differentially expressed genes from sorted immune cells were all upregulated, suggesting a polarized response after APS processing.

To explore the impact of APS processing on the immune cell types, we created different visualizations of the Nanostring data. The sample correlation heatmap of the Nanostring data highlights that regardless of APS processing, sorted T cells and granulocytes from both WBCs and APS have a strong correlation, while monocytes sorted from APS or WBCs have a low correlation (Fig. S2d). The sample correlation heatmap is a graphical representation of the data, where individual correlation values (represented as colors) are contained in a matrix with dark red corresponding to strong correlation (close to 1). This trend is further supported by the multidimensional scaling (MDS) plot of the expression differences between the sorted cell types, where T cells and granulocytes from APS and WBCs cluster closer together suggesting similarities in the gene expression, while monocytes from APS and WBCs do not cluster together (Fig. S2e). Coordinate 1 of the MDS plot is distinguished by cell type, while coordinate 2 stratifies samples according to the processing method. Of the 770 genes assayed, these data suggest that the APS processing alters monocyte gene expression more than T cells or granulocytes. This alteration may have implications on the monocyte activity during APS treatment.

### APS processing enriches M2-like dominant phenotype in monocytes

We investigated the monocyte phenotype after APS processing with flow cytometry. Here, changes in monocyte phenotype after APS processing were first approximated using macrophage polarization markers for M1- (CD80) and M2-like (CD163) phenotype, although the simplification of this classification does not address the heterogeneity and complexity that may exist. The mean fluorescence intensity (MFI) values for CD80 and CD163 were comparable among WBCs and APS samples. MFI values for CD163 were higher than MFI for CD80, suggesting an M2-like skewed phenotype for monocytes in WBCs prior to APS processing (Fig. 2a). MFI values for MHC-II were also comparable among WBCs and APS samples. APS processing resulted in a significant enrichment in CD163^+^ population in both classical monocytes from 158,000 ± 113,000 cells/mL to 1.74 million ± 1 million cells/mL. After APS processing, non-classical monocyte CD163^+^ population increased from 5200 ± 3000 cells/mL to 68,700 ± 66,000 cells/mL. Double-negative, CD80^−^CD163^−^ nonclassical monocytes were enriched from 10,300 ± 6500 cells/mL to 102,000 ± 114,000 cells/mL (Fig. 2b). CD163^+^ expression is induced by anti-inflammatory mediators and is important in resolving inflammation. The expression of CD163 may also suggest homeostatic, regulatory, or immune maintenance programming [19, 20]. Other monocyte subpopulations were present in smaller concentrations and were not significantly enriched after APS processing (Fig. S4a).

To further characterize the monocytes, we evaluated the percentage of CD163^+^ and CD80^+^ in classical (CD14^+^CD16^dim^) and non-classical monocytes (CD14^dim^CD16^+^). CD163^+^CD80^−^ was the dominant subtype in classical monocytes from APS and WBCs, 93.3% and 92.3%, respectively, while most non-classical monocytes were double-negative making up 58% and 66% of the population in APS and blood, respectively (Fig. 2c). There were few double-positive CD80^+^CD163^+^ and CD80^+^CD163^−^ classical monocytes and non-classical monocytes. APS processing resulted in a 16.5× ± 13.7 enrichment of a M2-like phenotype for classical monocytes (CD163^+^CD80^−^), while other subpopulations were enriched at lower amounts such as double negative CD80^−^CD163^−^ at 9× ± 6.4 or double-positive CD163^+^CD80^+^ at 1.1× ± 2.2, similar enrichment occurred in nonclassical monocytes (Fig. S4b). These findings suggest that APS processing further enriched the M2 polarization that was present among the donors and processing itself did not induce a phenotypic change. APS enriches CD163 in classical monocytes and non-classical monocytes by 17 and 12 times, respectively, the amount found in WBCs (Fig. 2c). To further characterize the monocytes, we evaluated the expression of antigen presentation marker, MHC-II. MHC-II^+^ monocytes 16% and 14% of classical monocytes in APS and in WBCs, respectively, while making up a larger percentage of non-classical monocytes, at 40% and 35% in APS and WBCS, respectively (Fig. 2d).

### GO analysis, Nanostring annotations, GSEA, and PPI network analysis of gene expression data show upregulation of antigen presentation and processing pathways in sorted monocytes

Further profiling analysis of the sorted monocyte (CD45^+^CD3^−^CD14^+^CD15^−^CD11b^+^CD11c^+^) population from APS and WBCs using the multiplex gene expression assay from Nanostring performed using the Rosalind platform found that there were 109 differentially regulated genes when comparing monocytes sorted from APS to monocytes sorted from WBCs; the top 50 differentially regulated genes are shown in Fig. 2e. To explore the predicted functional associations of the differentially expressed genes, a STRING protein-protein interaction (PPI) network was constructed only from the proteins that have physical interactions such as proteins that are part of a physical complex and visualized using Cytoscape 3.9.0, an open-source platform for complex network analysis in Fig. S4c [21]. The PPI network is highly connected with 107 nodes (differentially expressed genes) and 268 edges (interactions between nodes). We then used tools for knowledge discovery to predict important genes in the network for additional insights into how gene expression changes due to APS processing could impact biological processes or pathways. To determine essential genes in the network, centrality measures were quantified using CytoNCA 2.1 [22]. Based on the top-ranked centrality measures for subgraph centrality, betweenness centrality, and closeness centrality, essential genes in the network are *Fyn*, *Jak1*, *Jak3*, *Mapk1*, *Stat1*, *Stat3*, and *Syk.* These genes are represented in green in the PPI network in Fig. S4c.

To identify highly interconnected regions in the PPI network nodes based on the connectivity degree of networks, the MCODE, molecular complex detection algorithm Cytoscape plugin was used [23]. The module analysis identified the most significant module containing 19 nodes, *Jak1*, *Jak3*, *Stat3*, *Stat1*, *Fyn*, *Stat6*, *Stat5b*, *Il4r*, *Il6r*, *Il6st*, *Hla-dra*, *Hla-c*, *Hla-dpb1*, *Hla-g*, *Hla-b*, *Hla-a*, *Hla-e*, *Tapbp*, *Tap1*, with 69 edges. The subnetwork of this module is represented in orange with the first neighbors represented in blue as depicted in Fig. S4d. The essential genes identified by the centrality measures were all in the significant module. Notably, 12 of the 19 are in the top 50 differentially expressed genes.

To further explore potential changes in the biological function and key pathways, Gene Ontology (GO) analysis was performed using STRING. Gene Ontology (GO) analysis of all significantly differentially regulated genes found that for the category of biological processes, sorted monocytes from APS had functional enrichment related to the following GO terms relevant to tissue repair: “IL-4-mediated signaling pathway and antigen processing” and “presentation of endogenous peptide via MHC class I via ER pathway, TAP-independent.” The molecular function category found enrichment in terms related to antigen processing such as TAP binding and TAP2 binding. The cellular component category found enrichment in macrophage migration inhibitory factor receptor complex. Functional enrichment using the protein domains (Pfam) database found enrichment in the following domains: MHC-I C-terminus and STAT protein (protein interaction domain, DNA-binding domain, and all alpha-domain).

Additional pathway collections were explored for enrichment analysis using the Rosalind platform (*p*_adj_ < 0.05). MySigDB Pathway Collection found several significantly enriched terms associated with antigen processing and signaling processes (Fig. 2f). Enrichment was found for the following MSigDB REACTOME terms, Class I MHC mediated antigen processing presentation (*Ubc*, *Psmb8*, *Tap1*), antigen processing cross-presentation (*Psmd7*, *Ly96*, *Hla-g*), antigen presentation folding assembly and peptide loading of class I MHC (*Hla-g*, *Hla-c*, *Hla-e*), and adaptive immune system (*Sell*, *Psmd7*, *Ly96*). The Wiki pathways showed enrichment of proteosome degradation, IL-4 signaling pathway (*Fos*, *Stat3*, *Tyk2*), IL-2 signaling pathway (*Stat1*, *Fyn*, *Syk*), and oncostatin M signaling pathway (*Prkcd*, *Jak1*, *Jak3*).

We then performed gene set enrichment analysis using Nanostring Annotations on the Rosalind platform and found enrichment of terms related to cytotoxicity (*Hla-a*, *Hla-b*, *Hla-b*), antigen processing (*Tap1*, *Tapbp*, *Psmb9*, *Psmb7*), transporter functions (*Fyn*, *Itgam*, *Cd47*, *Cd44*), and cell cycle (*Cxcr4*, *Tnfsf10*). The overall differential expression of each gene set (directed enrichment score) showed 4 times differential expression in APS sorted monocytes compared to blood (Fig. 2g).

Altogether, the pathway analyses identified antigen processing-associated gene sets or terms in all the databases and collections used. *Tap2*, an antagonist peptide of TLR-4, had analgesic and anti-inflammatory effects in a monoiodoacetate (MIA)-induced rat model of OA that resulted in decreased cartilage loss [24]. *Tap2* can reduce ROS in the arthritic joint. Pathways and terms identified from the analyses employed suggest an association with anti-inflammatory functions in monocytes (*Ifi16*, *Stat3*, *Il10ra*, *Cxcr4*, *Cd63*, *Cd47*). *Stat3*, *Il10ra*, and *Cxcr4* were in or the first neighbors of the densest region of the PPI network and had more than 4 times fold change in expression.

### Lymphocytes are enriched and maintain phenotypic subsets after APS processing

**Fig. 3:**
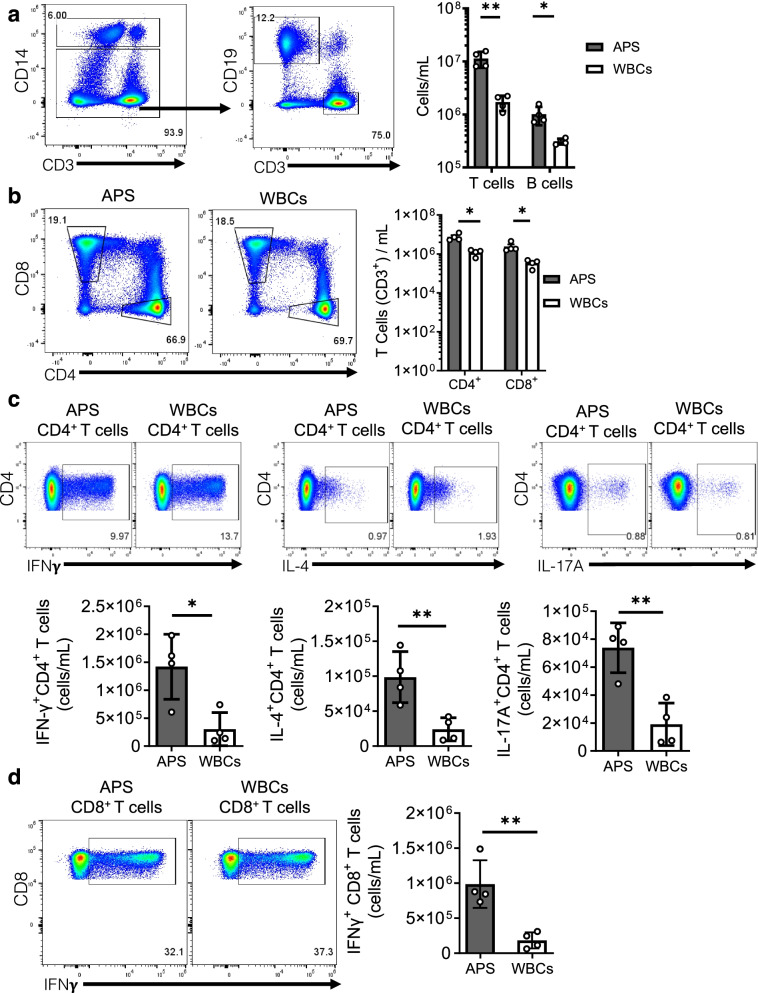
Lymphocytes are enriched and maintain cytokine production functionality after APS processing.** a **Gating schematic of T cell (CD3^+^) and B cell (CD19^+^) surface staining panel. Quantification of T and B cell concentration in APS and WBCs on a log scale. **b** Flow cytometry plot of CD8^+^ and CD4^+^ T cells and quantification of CD4^+^ and CD8^+^ T cell concentrations in APC and WBCs on a log scale. **c** Flow cytometry plots of intracellular cytokine staining results for IFN𝛄^+^, IL-17A^+^, and IL-4^+^ CD4^+^ T cells in APS and WBCs and quantification of IFN𝛄^+^, IL-17𝛂^+^, and IL-4^+^ CD4^+^ T cell concentration in APS and WBCs. **d** Flow cytometry plots of intracellular cytokine staining results for IFN𝛄^+^CD8^+^ T cells in APS and WBCs and quantification IFN𝛄^+^CD8^+^ T cell concentration in APS and WBCs. All data are mean with ± SD. Multiple unpaired *t*-test using Holm-Sidak method with set *p*-value threshold, alpha = 0.05 for **a**–**c**. Unpaired *t*-test with two-tailed *p*-value, alpha = 0.05 (**d**). *p < 0.05, **p < 0.001

We used flow cytometry to investigate the impact of APS processing on lymphocyte concentration. CD3^+^ T cells were more prevalent than CD3^−^CD19^+^ B cells. APS processing significantly increased the concentration of T cells and B cells from 1,710,000 ± 547,000 cells/mL and 310,000 ± 40,000 cells/mL in WBCs to 11 million ± 3,800,000 cells/mL and 1 million ± 384,000 cells/mL after APS processing, respectively (Fig. 3a). CD4^+^ T cells were the dominant T cell subtype, then CD8^+^. CD4^+^ and CD8^+^ T cell subsets were significantly increased from 1,210,000 ± 420,000 to 8 million ± 3,300,000 cells/mL and 343,000 ± 143,000 to 2,430,000 ± 1,380,000 cells/mL, respectively, after APS processing (Fig. 3b).

We then used intracellular staining to investigate phenotypes of T cells. After APS processing, CD4^+^ and CD8^+^ T cells maintain the potential to express cytokines as shown by intracellular staining after stimulation with PMA/ionomycin, gating strategy shown in Fig. S5. CD4^+^ IFNγ^+^, IL-4^+^, and IL-17A^+^ were significantly enriched after APS processing compared to WBCs. CD4^+^IFNγ^+^ T cells were enriched from 303,000 ± 297,000 to 1.42 million ± 582,000 cells/mL, CD4^+^IL-4^+^ T cells increased from 24,000 ± 17,000 to 99,000 ± 36,000 cells/mL, CD4^+^IL-17A^+^ T cells 19,000 ± 15,000 to 74,000 ± 18,000 cells/mL (Fig. 3c). CD8^+^IFNγ^+^ T cells were also significantly enriched after APS processing, with an increase from 184,000 ± 115,000 to 990,000 ± 340,000 (Fig. 3d). These findings suggest that the functionality of the WBCs is maintained after APS processing and that the second most abundant immune cell subpopulations in APS, T cells, could be an additional source of bioactive factors.

### Immune cell fraction persists after APS treatment in RAG KO OA model

**Fig. 4:**
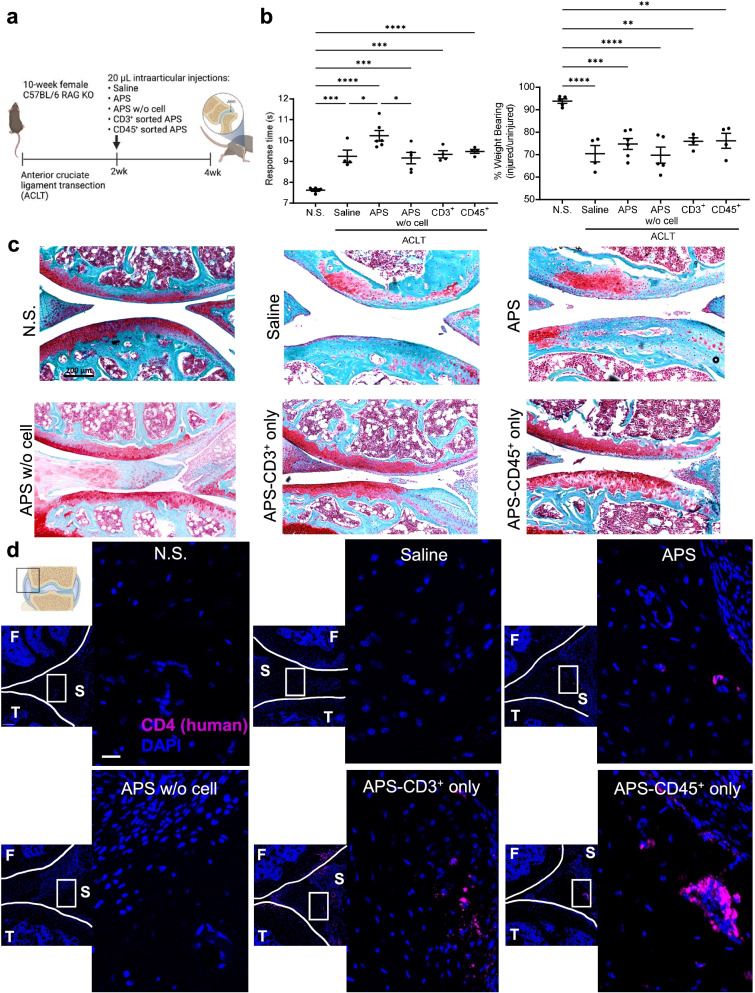
Immune cell fraction from APS treatment persists in RAG KO OA model. Ten-week female C57BL/6 RAG KO mice underwent unilateral ACLT surgery and treatment 2 weeks later with saline (control), human APS, APS without cellular component (denoted as APS w/o cell), sorted CD3^+^ cells from APS (denoted as CD3^+^ or APS-CD3^+^ only), or sorted CD45^+^ cells from APS (denoted as CD45^+^ or APS-CD45^+^ only) (*n* = 5/group), and no surgery (N.S.) control. **a** Study design. **b** Average response time after treatment. Percentage of weight placed on the operated limb versus the contralateral control limb (data are mean with ± SEM). **c** Representative brightfield images of safranin-O fast green staining of joint (× 20 magnification). Scale bar = 200 μm. **d** Immunofluorescence staining of human CD4^+^ cells (pink) that does not react with mouse and DAPI counterstain (blue) in the synovium of the operated joints 2 weeks after injection with APS, APS without cellular component, CD3^+^ sorted from APS, CD45^+^ sorted from APS, saline, and no surgery (N.S.) control (× 20 magnification). Scale bar = 200 μm for joint and for zoomed-in ROI scale bar = 20 μm. Ordinary one-way ANOVA with Tukey’s multiple comparison tests for **b**. *p < 0.05, **p < 0.01, ***p < 0.001, ****p < 0.0001

To explore how different components of human-derived APS can impact local response to an injury, we used a post-traumatic osteoarthritis model in 10-week female C57BL/6 RAG KO mice. Two weeks after inducing unilateral OA via ligament transection, experimental treatment groups (*n* = 5/group) were injected with either whole human APS, 3000 sorted CD45^+^ cells from APS, 3000 sorted CD3^+^ cells from APS, or the acellular portion of APS (containing soluble proteins only). Saline injections and no surgery groups were used as controls. Injections of 20 μL for each experimental group ensured the maintenance of the integrity of the joint capsule and localization of the APS near the joint (study design in Fig. 4a). Two weeks after injections, for each experimental group, the animals were harvested for histology of the joints (*n* = 1/group) and qRT-PCR of the inguinal lymph nodes (*n* = 5/group) and the remainder of the joints (*n* = 4/group). Weight-bearing measurements were not restored for any of the treatment groups when compared to no surgery controls. Significantly longer response times in the APS-treated group suggest that the group could be experiencing more pain than the saline and APS without cell groups (Fig. 4b). Representative safranin-O staining (*n* = 1/group) is provided in Fig. 4c to observe proteoglycan content in the joint.

We then investigated the survival of the cell fraction of APS after injections. Immunofluorescence staining confirmed the persistence of the cell fraction of APS 2 weeks after single injections. The presence of human positive CD4 cells in the synovium of the APS treatment groups with cells (APS, CD3^+^ sorted from APS, and CD45^+^ sorted from APS) demonstrates cell persistence and the durability of the cell fraction that may be important for APS treatment outcomes (Fig. 4d). Tissue-resident immune cells can be long-lived. Lymphocytes can persist for weeks to even years as is the case for memory T cells. The injected immune cells persist beyond the lifespan of platelets contributing to the microenvironment well beyond the initial injection and the biological activity of proteins in APS.

To investigate the potential systemic effects of the treatment, the gene expression of pro-inflammatory and anti-inflammatory cytokines in the inguinal lymph node that drains lymphatics from the injured articular joint were evaluated. *Il1b*, *Il10*, *Ifng*, and *Il4* did not significantly change among the experimental groups in the draining inguinal lymph nodes suggesting that the injections did not polarize the systemic response (Fig. S7a). IL-17 cytokines have demonstrated pathophysiology in osteoarthritis in preclinical and clinical studies [25]. Quantification of *Il17f* mRNA in the draining inguinal lymph node increased in some mice in the saline and APS without cell treatment groups compared to the other groups, although did not reach statistical significance (Fig. S7a). *Il17a* was not detected in the joint or lymph node for all groups.

To investigate the local effects of the treatment, the gene expression of relevant genes of the whole joint were explored. A broad-spectrum MMP inhibitor important for reducing cartilage degradation, *Timp1*, had significantly increased expression in the joint for all treatment groups compared to no surgery control. Regulatory cytokine, *Tgfb1*, had significantly increased expression in the joint for APS, APS without cell, and CD3^+^ sorted from cells from APS treatment groups compared to no surgery control. *Tgfb2* expression was not significantly differentially expressed among treatment groups. *Il17b* expression in the joint did not significantly change among the treatment groups*.* Additionally, the pro-inflammatory cytokine expression in the joint, *Tnfa*, metalloproteinase *Mmp12*, and *Ddr1*, known to control MMP-13 expression during chondrogenesis, had no significant difference among the groups (Fig. S7b)*.* Pro-inflammatory cytokine *Il1b* expression was significantly increased for the CD3^+^ sorted from the APS group when compared to all groups.

### The cell fraction of APS is the main source of anti-inflammatory IL-1Ra cytokine and is enriched after APS processing

APS can be prepared from a broad range of OA patients with consistent preferential enrichment of anti-inflammatory cytokines [4]. The pleiotropic nature and kinetics of cytokines in the wound healing cascade require further understanding of the OA pathology and treatment. To evaluate the concentration of prominent cytokine and growth factors studied in the wound healing cascade, we performed Luminex cytokine assays on the cellular compartment (lysates) and the soluble fraction (plasma) of APS and blood. All white blood cell lineages produce cytokines. Once activated, platelets release stored cytokines and chemokines. We did not distinguish the Luminex results by cell type. Based on the literature, we can hypothesize what cell types may be contributing to the source of relevant bioactive factors. In APS, IL-1Ra was the most abundant protein (103,000 ± 50,000 pg/mL), followed by IGF-1 (93,000 ± 17,000 pg/mL), and TGF-β1(15,000 ± 8000 pg/mL). IGF-1 is a growth hormone mainly produced by the liver and then released into circulation [26]. TGF-β1 is a pleiotropic cytokine produced by all white blood cells [27]. IL-1β and TNF-α are known pro-inflammatory cytokines that promote OA pathophysiology and are mainly produced by macrophages [28]. IL-1β and TNF-α can also be released by activated platelets [29]. TNF-α is also secreted by other immune cells such as monocytes, T cells, NK cells, and NKT cells [30]. IL-1β and TNF-α were present at low levels, 57 ± 5 pg/mL and 40 ± 27 pg/mL, respectively, in APS (Fig. S6a). Other studies have noted the presence of remarkably low concentrations of IL-1β in APS [7]. Another study characterizing the cytokine profile of APS did not detect TNF-α [4]. The non-cellular soluble protein fraction significantly enriched other anabolic cytokines such as TGF-β1 and IGF-1 by 5× and 2×, respectively (Fig. S6b). The balance between IL-1Ra and IL-1β has a critical role in the homeostasis of inflammation. It is important to note the concentrations of the cytokines present. Although pro-inflammatory cytokines like IL-1β and TNF-α are present in APS, anti-inflammatory cytokines are present at concentrations 4–5 orders of magnitude higher.

Among anti-inflammatory cytokines enriched in APS, the assay revealed a significant increase, 17× fold change, of IL-1 receptor antagonist (IL-1Ra) specific to the cellular fraction (lysates). The significant increase in IL-1Ra is comparable to other studies characterizing APS cytokine enrichment [4]. IL-1Ra was not detectible in the plasma fraction. This is notable because previous studies have found that APS has a 5.9-fold increase in IL-1Ra and 5-fold increase in WBCs compared to whole blood [4]. Findings from the first in-human clinical trial with a single injection of APS found that subjects with high concentrations of WBCs and IL-1Ra:IL1-β ratio greater than 1000 in APS were more likely to respond to the APS therapy than the total study population [31]. WBC concentration was significantly correlated with IL-1Ra in APS, as predicted given that the common isoforms of IL-1Ra are generally found inside of the cell [32]. The intracellular isoform of IL-1Ra is primarily produced by neutrophils and monocytes [33–35]. Neutrophils are the dominant immune cell type in the final product of APS and are likely the main source of potent anti-inflammatory intracellular IL-1Ra. The high IL-1Ra:IL1-β ratio for the healthy volunteers in the present study suggests that they would respond to APS injections. The Luminex cytokine assay confirmed that the cell fraction of APS is the major source of IL-1Ra, as shown in previous studies [7]. Of the analytes studied, IL-1Ra was the only analyte detected in the cell fraction. IGF-1, IL-1β, TGF-β1, TGF-β2, and TGF-β3 were not detected in the cell fraction of APS. TGF-β3 was not detected in either APS or blood. A small concentration of TNF-α (4.7 pg/mL) was detected in one of the APS cell fraction samples. Notably, TGF-β2 (386 ± 38 pg/mL) was detected in the blood plasma fraction but not in the plasma or cell fraction of APS (Fig. S6a).

## Discussion

The main finding of this study is that APS processing alters the immune cell composition found in blood and APS processing induced significant differential gene expression dependent on cell type that may be important for OA treatment outcomes. Here, we profiled the immune population in APS, an orthobiologic therapy for the management and treatment of knee osteoarthritis, by defining the immune cell type composition and phenotype.

Blood derivatives are complex mixtures of varying degrees of bioactive factors and cells, and whose mode of action in treating musculoskeletal pathologies is not fully understood. Clinical studies have shown that the autologous transfer of orthobiologics with immune cells into an arthritic joint may contribute significantly to the therapeutic effect of APS [4, 14, 31]. In the first in-human clinical trial for APS, high concentrations WBC and IL-1Ra were associated with responders and improved therapeutic outcomes. These findings suggest that the cellular component of APS has an important role in its therapeutic effect. Yet, leukocyte inclusion in PRP therapies has not been established due to limited RCTs. For clinical studies that use PRP-containing leukocytes, most indicate whether leukocytes are present or not, but do not investigate cell composition changes due to processing or define the immune subtypes present. In this study, we quantitatively assessed the changes in immune cell populations after APS processing compared to WBCs. We found that CD45^+^ cells are viable and highly concentrated in APS, with neutrophils and T cells being the most abundant immune cell types.

Immune cell phenotype and activation state are critical for OA disease status and progression. Known cell phenotypes have specific functions and produce cytokines and chemokines to influence the microenvironment. Resident macrophages play an important role in normal joint physiology and in OA progression. Endogenous DAMPs inherent in the catabolic microenvironment contribute to inflammasome activation and macrophage polarization. Infiltrating monocyte-derived macrophages have been shown to promote cartilage inflammation and degeneration [36]. In this study, we explored monocyte phenotypes after APS processing. Flow cytometric analysis found that the dominant monocyte population was classical monocytes in APS and WBCs. CD163^+^ expression was more dominant than CD80^+^ in both classical and non-classical monocytes, with CD163^+^ classical monocytes being the most abundant monocyte subtype in APS. APS processing enriched the anti-inflammatory CD163^+^ skewing that was already present in WBCs. We also explored potential changes in biological function and key pathways in sorted monocytes after APS processing using the Nanostring gene expression assay. Sorted monocytes from APS had significant differentially expressed genes related to antigen processing and anti-inflammatory functions compared to sorted monocytes from WBCs. Module analysis and centrality measures of the PPI network constructed from the differentially expressed genes highlighted many of the genes that were in enriched terms and gene sets such as *Tap1*, *Tapbp*, *Hla-g*, and *Stat3*.

PRP therapies can contain both pro-inflammatory and anti-inflammatory growth factors at varying concentrations. Different formulations of PRP can have inhibitory effects on clinical outcomes. APS processing method results in a robust mixture of antagonists of pro-inflammatory cytokines and concentrated growth factors with low levels of pro-inflammatory factors. Previous studies have recognized donor variability in APS composition and its impact has been studied in vitro. Independent of the donor variability, APS consistently reduced IL-1β driven IL-8 and TNF-α production in human THP-1 monocyte cells [7]. Of the biomolecules evaluated in this study, anti-inflammatory cytokines such as IL-1Ra and IGF-1 were present at higher concentrations than pro-inflammatory cytokines such as IL-1β and TNF-α. TGF-β1 had a five times fold increase in APS when compared to blood. We did not investigate the source of the biomolecules assayed by cell type. These findings are consistent with previous studies that have shown that APS has high concentrations of anabolic growth factors such as platelet-derived growth factor (PDGF) and insulin-like growth factor-1 (IGF-1) [4]. TGF-β1 and other growth factors are important during the initial stages of wound healing. Monocyte-derived macrophages are a source of growth factors such as PDGF, TGFβ-1, and FGF. In vitro studies have found that TGF-β1 increases collagen synthesis and deposition particularly through macrophage-fibroblast crosstalk [37]. TGF-β1 is generally regarded as anti-inflammatory and promotes alternative macrophage activation [38]. In the RAG KO study, *Tgfb1* significantly increased in the joint after APS treatment. WBCs are a major source of antagonists of pro-inflammatory cytokines such as IL-1Ra in the body. Notably in this study, IL-1Ra was only found in the cell fraction of APS. IL-1Ra is an important suppressor of inflammation that competitively binding to IL-R1 thereby inhibiting IL-1α and IL-1β to IL-1RI leading to a decrease in cartilage and bone destruction. IL-1Ra has native biological functions such as attenuating IL-1-inducible gene expression and inhibiting IL-1-induced IL-6 and IL-8 production [39, 40]. In the RAG KO study, a MMP inhibitor, *Timp1*, significantly increased expression in the joint after APS treatments when compared to no surgery control. This is in concordance with in vitro APS studies that found inhibition of destructive proteases such as reduction of MMP-13 from IL-1β and TNF-α stimulated chondrocytes [41].

Immunofluorescence staining of human cells confirmed persistence and survival after APS injections in the RAG KO in vivo study. These results suggest that the cellular component of APS may provide a more durable treatment than the soluble protein fraction alone due to the long-lived nature of some tissue resident leukocyte subtypes, such as T cells. T cells were the second most abundant immune cell subtype after APS processing. CD4^+^ T cells were more abundant than CD8^+^ T cells after APS processing and both subtypes maintained the propensity to produce cytokines. Immune cells can provide extended release of bioactive factors. This is an important consideration given that platelets are thought to deplete the bioactive factors in alpha-granules within an hour of activation [42]. Additionally, as demonstrated by Mariani et al., the leukocyte cell fraction can influence the extent of degranulation and thus the concentration of bioactive factors present [16]. Younger and thereby more dense platelets are often in the buffy coat or WBC fraction after common density gradients centrifugation techniques used in PRP processing [42]. This suggests that PRP with leukocytes also contain more platelets with denser alpha granules than PRP without leukocytes. In one study, platelet concentration was higher in leukocyte-rich PRP than in pure PRP regardless of centrifugation speeds [43]. Non-platelet cells in PRP are required for normal platelet functions such as thrombin generation, growth factor release, and clot retraction [44]. The 3-year follow-up for the multicenter double-blind randomized saline-controlled trial investigating the effects of a single intra-articular injection of APS found that for patients with mild to moderate knee OA, APS was safe, and patients maintained significant pain improvement [15]. Meta-analysis of RCTs comparing PRPs to alternative injections found that PRPs reached clinical significance at 6-month and 12-month time points suggesting the durability of PRP [9]. These preliminary findings highlight the potential durability of orthobiologics and future investigation should identify the durable aspects of the therapy, particularly exploring the durability of the cellular component.

Classically activated M1 macrophage phenotype contributes to OA progression by producing pro-inflammatory mediators that modulate synovial fibroblasts, chondrocytes, and other immune cells. A study evaluated immune cell infiltration composition in human OA tissue and normal control subjects’ microarray data to identify diagnostic markers based on gene expression profiles and found an increase in M1 macrophage infiltration and a decrease in mast cell and neutrophil infiltration in OA tissue [45]. In another study, a higher M1/M2 macrophage ratio has been found in OA patient’s peripheral blood and synovial fluid compared to healthy controls. The M1/M2 macrophage ratio was significantly associated with the Kellgren-Lawrence classification of osteoarthritis [46]. Inhibiting inflammation mediated by macrophages has been suggested as a therapeutic strategy for OA [5]. In preclinical studies, targeting macrophages has resulted in differential outcomes in OA. One study with transgenic mice with enhanced M1 macrophages or M2 macrophages found disease progression with M1 macrophages and attenuation with M2 [47]. Another study found that depleting M1 and M2 macrophages in obese Fas-induced apoptosis-transgenic mice did not reduce OA progression [48]. There is a spectrum of macrophage phenotypes involved in OA and depletion of one or promotion of the other may not be therapeutically relevant. These data indicate a multifunctional therapy is needed and that therapies targeting macrophages alone is not sufficient. APS is a complex mixture of anti-inflammatory cytokines, growth factors, and immune cells, whose components are acting on various aspects of OA pathophysiology.

The limitations of the present study are recognized. We evaluated the impact of APS processing using healthy volunteers instead of OA patients. Previous studies have demonstrated APS processing in all patients results in anabolic and anti-inflammatory product irrespective of OA status or age [4]. WBCs were used as a control to compare APS processing instead of whole blood. WBC isolation is not an exact representation of whole blood, yet WBCs serve as an appropriate control because we wanted to evaluate the immune cell composition. Density gradients used to isolate WBCs capture most of the immune cells from the whole blood. Additionally, the original whole blood sample volumes were used when calculating cell concentrations. PRP contains hundreds of bioactive molecules and only a few were evaluated here. PRP therapies are unique in that the therapy has widespread applicability, yet specific formulations with enrichment of certain cell types/subtypes and bioactive factors may be needed for different pathologies. Thus, it is critical to study more bioactive factors specific to different applications. Mariani et al. evaluated PRP biomolecules not commonly evaluated in PRP characterization studies such as RANTES/CCL-5, MCP-3/CCL-7, and PF-4/CXCL-4 and found differences in concentrations among different PRP preparation methods [16]. Another limitation of the study was the small sample size used for Nanostring gene expression analysis (*n* = 4). The small sample size limited the statistical power of the study and did not allow for the exploration of potential donor variability related to gene expression. However, we were able to find a polarizing change in gene expression in monocytes after APS processing, and many genes related to similar biological processes demonstrating upregulation of antigen processing-associated genes and anti-inflammatory functions that likely contribute to the therapy. We did not identify what aspects of the processing technique causes preferential enrichment and gene expression changes in immune cells. Future studies investigating immune cell changes due to specific parts of APS processing will provide further understanding to optimize next-generation orthobiologics. We were not able to assess the immune effects of APS in the immunocompromised RAG KO murine model. We demonstrated the durability of the immune cell fraction of APS suggesting the cell fraction may play a role in the therapy beyond the initial injection and the immune cells in part may explain the durability seen clinically. We did not study the maintenance of APS cells and their phenotypes after injection in the OA murine model. Additional studies characterizing the immune cell phenotype after injections are needed to determine how the APS cell fraction directly contributes to the clinical utility of APS treatment, although this may differ between preclinical models and clinical populations. Furthermore, there is a reciprocal influence of the injected cells and the microenvironment. The injected immune cells in the APS can modulate the local environment by producing specific paracrine factors and cytokines and vice versa the cells and extracellular matrix of the local milieu can in turn influence the APS cells. Multiple studies demonstrate that the circulating bioactive factors and cells in the blood are harnessed for regenerative processes when an acute injury occurs. Blood-derived orthobiologics are thought to promote similar regenerative processes when locally injected into arthritic joints, although the mechanism of action is not fully understood. RAG KO mice do not produce mature T and B cells. Although this feature of RAG KO mice is important to allow us to inject human cells into the mice without immunogenic reactions, it does not allow us to make any meaningful conclusions about the therapeutic effects of APS in this model. It also does not allow us to meaningfully examine APS phenotype changes considering the full context of the pathophysiology of OA that includes the adaptive immune system. Future studies would require immune competent and autologous APS to draw conclusions about which immune cell types are important for functional healing, how the APS cell phenotype may change once injected into an arthritic joint and the contribution of these cells to the therapeutic effects of APS. Nonetheless, we were able to explore the survival of APS cells after injection in the in vivo model.

Elucidation of the therapeutically active components of PRP is important in informing future development in processing techniques that optimize durability and potential selective enrichment of therapeutic components. There are known differences in the immune response due to various factors such as age and sex. Additional investigation of how age, sex, and patient heterogeneity broadly may impact the immune cells and resulting mechanism of action after PRP processing is needed. Further understanding of the therapeutically active components of PRP such as long-lived white blood cells will also help predict whether a patient will respond to the therapy based on their PRP composition ahead of treatment to better inform treatment approaches. Bioactive PRP components not explored in this study such as extracellular vesicles are present in PRP due to platelet shedding, are immunomodulatory, and support low-grade thrombin generation [49]. Previous studies have suggested the role of apoptotic microparticles from platelets in polarizing monocytes to M2 pro-healing macrophages (CD14^+^CD16^+^CCR5^+^CXCR4^++^) [50]. Future work related to understanding the contribution of other components of PRP such as the extracellular vesicles is needed. Further characterization of APS will be critical in gaining clinical insights into a specific mode of action of the treatment, particularly on understanding what is contributing to the durability of the treatment. Determining optimal composition, volume of injection, and number and/or frequency of injections needed will help establish standard guidelines for applications of orthobiologics in the treatment of knee OA.

The local injection of PRP at a diseased site can mediate pro-inflammatory processes and reduce the catabolic environments associated with knee osteoarthritis to restore homeostasis and promote repair. Although PRP has widespread use, there is limited standardization in its composition in terms of the cellular and soluble protein fractions. There have been limited RCTs to elucidate the therapeutic contributions of each component in PRP. The varying composition of PRP due to different processing techniques and lack of standardization contributes to the limited knowledge of the therapeutically relevant components. Understanding the processing effects on orthobiologic composition and concentration of cell phenotypes and bioactive factors required for favorable therapeutic outcomes are important to improve reproducibility and the predictive capacity of the treatment. Although platelets are purported to be the dominant therapeutic factor, the mechanism of action for PRP is not fully understood and the role of the immune cell fraction not fully interrogated. Our findings suggest that APS processing alters the immune cell composition and phenotype. The persistence of the APS cell fraction after injections suggests the cell fraction may explain the treatment durability of APS seen clinically. Future work defining therapeutically relevant PRP components and further evaluation of PRP therapies broadly in RCTs is needed for next-generation orthobiologics and meaningful consistent clinical outcomes.

## Materials and methods

### APS processing

Blood samples were obtained from 4 donors (100–120 mL). The blood samples were collected in heparinized tubes and split for WBC collection, APS processing, and 1 mL of whole blood was allocated for Luminex assay control. Samples from all donors were prepared on the same day. Briefly, for APS processing, the blood (40–55 mL) was drawn into a 60-mL syringe with 5 mL of anticoagulant citrate dextrose solution formula A (ACD-A) and processed with the single-use nSTRIDE® APS kit (Biomet Biologics, Warsaw, IN) to obtain APS. The first part of the processing separates the cellular and platelet components using the nSTRIDE® Cell Separator. The second part of the process uses the nSTRIDE® Concentrator to concentrate the cell solution using polyacrylamide absorbent beads to produce APS [31].

### WBC processing, flow cytometry, and FACS fluorescence-activated cell sorting of APS

All samples were brought up to 40 mL in HANK’s balanced salt solution, HBSS, and prepared using either Ficoll-Paque PLUS solution (Pharmacia LKB, Uppsala, Sweden) density gradient centrifugation or with red blood cell lysis using ammonium-chloride-potassium lysis buffer (Quality Biologica) according to manufacturer’s protocol. For intracellular staining, cells were stimulated with Cell Stimulation Cocktail plus protein transport inhibitors (eBioscience). Cells were subsequently washed and stained with surface markers. Then, cells were fixed and permeabilized using Cytofix/Cytoperm (BD), followed by staining with intracellular markers. Flow cytometry experiments were performed using the Attune NxT flow cytometer (Thermo Fisher Scientific). FACS was performed using the FACSDiva (BD Biosciences) or FACSAria II (BD Biosciences). Cells were stained using the antibody panels listed in Additional file 1: Table S1. For each experiment, all donor samples were recorded on the same day. Total cell concentrations were quantified manually and based on the original volume of the whole blood sample used. Frequencies of live cells from flow cytometry were used to estimate cell concentrations for each desired cell subtype by multiplying the frequency of live cells for that subpopulation and the manually quantified cell concentration of the entire sample.

### Nanostring gene expression analysis

Immune cell fractions were sorted using FACS from APS and blood (granulocytes, T cells, and monocytes) and were used to isolate RNA for Nanostring gene expression profiling. Samples were stored in 750 μL TRIzol LS Reagent (Invitrogen) at − 80 °C prior to assay. The RNeasy Plus Micro kit (Qiagen) was used according to the manufacturer’s protocol for RNA isolation. Samples were hybridized with the Reporter CodeSet according to the manufacturer’s protocol. Gene expression profiles were obtained using the Nanostring nCounter analysis system using the Nanostring PanCancer Immune profiling CodeSet (770 genes). Data were analyzed by ROSALIND® (https://rosalind.onramp.bio/), with a HyperScale architecture developed by ROSALIND, Inc. (San Diego, CA). Read distribution percentages, violin plots, identity heatmaps, and sample MDS plots were generated as part of the QC step. Normalization, fold changes, and *p*-values were calculated using criteria provided by Nanostring. ROSALIND® follows the nCounter® Advanced Analysis protocol of dividing counts within a lane by the geometric mean of the normalizer probes from the same lane. Housekeeping probes used for normalization were selected based on the geNorm algorithm as implemented in the NormqPCR R library1. The abundance of various cell populations was calculated on ROSALIND using the Nanostring cell type profiling module. ROSALIND performs a filtering of cell type profiling results to include results that have scores with a *p*-value greater than or equal to 0.05. Fold changes and *p*-values were calculated using the fast method as described in the nCounter® Advanced Analysis 2.0 User Manual. *p*-value adjustment was performed using the Benjamini-Hochberg method of estimating false discovery rates (FDR). Clustering of genes for the final heatmap of differentially expressed genes was done using the partitioning around medoids (PAM) method using the fpc R library2 that takes into consideration the direction and type of all signals on a pathway, the position, role, and type of every gene, etc. Hypergeometric distribution was used to analyze the enrichment of pathways, Gene Ontology, domain structure, and other ontologies. The topGO R library3, was used to determine the local similarities and dependencies between GO terms to perform Elim pruning correction. Several database sources were referenced for enrichment analysis, including Interpro4, NCBI5, MSigDB6,7, REACTOME8, and WikiPathways9. Enrichment was calculated relative to a set of background genes relevant to the experiment [51–59]. Statistical significance limit adj *p* < 0.05 with fold change threshold set to ± 1.5. The sample correlation heatmap was created using the ComplexHeatMap R package [60]. PPI network of the differentially expressed genes was created using the STRING Database (version 11.5, https://string-db.org/) [61]. STRING PPI network analysis was employed using the STRING Database. The STRING PPI network was visualized using Cytoscape 3.9.0 [21]. A Cytoscape plugin, CytoNCA 2.1, was used to quantify centrality measures [22]. Default settings for the Cytoscape plugin, MCODE, molecular complex detection algorithm, were used to identify highly interconnected regions of the PPI network [23].

### Luminex assay

Two milliliters APS and 1 mL whole blood controls from each donor (*n* = 4) were separated into plasma (soluble fraction) and cellular components (lysates) by centrifugation at 1000*g* for 5 min. The plasma and cell lysates were collected and stored at − 80 °C prior to assay. The Luminex assays were performed by the human immunology core at Johns Hopkins using the Luminex xMAP technology. The assays were conducted following HIC SOP-25 “Millipore Cytokine Assay” with modifications to match specific vendor guidelines for the corresponding Milliplex magnetic bead panel chemokine/cytokine. The samples were run in triplicate wells. Samples for IGF-1 were diluted at 1:60, for TGFβ1,2,3 samples were diluted at 1:30. For IL-1β, TNF-α, and IL-1Ra, samples were not diluted. Standard curves were generated for each analyte to determine the concentration for each sample. Sample dilutions from APS processing and assay dilutions were accounted for in the reported final concentration calculations.

### Surgical procedures

The post-traumatic osteoarthritis murine model in 10-week female C57BL/6 RAG KO (The Jackson Lab) mice (*n* = 5/group) was employed by unilateral anterior cruciate ligament transection. Two weeks after the onset of OA, treatment groups were injected intra-articular with 20 μL of saline, human APS, APS without cellular component, CD3+ sorted cells from APS, or sorted CD45+ from APS. The APS was processed from 3 donors and pooled. Animals were harvested two weeks after injections for qRT-PCR of the draining lymph nodes and joints and histological analysis of the joint.

### Real-time quantitative PCR assay

Total RNA was extracted using TRIzol (Invitrogen) according to the manufacturer’s protocol from whole lymph nodes ipsilateral to injury or injury-bearing whole joint with excessive muscle/fat tissues removed. RNA purification was performed using the RNeasy Plus Mini kit (Qiagen). Briefly, for mRNA detection, 2.5 μg of mRNA was synthesized into cDNA using Superscript IV VILO Master Mix (Invitrogen). Quantitative real-time qRT-PCR was carried out using Taqman primers (Thermo Fisher Scientific) and a StepOnePlus Real-time PCR System (Life Technologies) at 100 ng/well cDNA in a total of 20 μL PCR reaction volume. Relative gene expression was calculated by the Livak Method using ΔΔCt. The ΔCt was calculated using the reference gene RER-1 and experimental groups were normalized to no surgery controls.

TaqMan primers were used for detection in the joint and inguinal lymph nodes. The IDs are the following: *Rer1*: Mm00471276, *Ifng: Mm01168134_m1*, *Il1b*: Mm00434228, *Il4*: Mm00445259, *Il10*: Mm01288386, *Il13*: Mm00434204, *Il17f: Mm00521423_m1*, *Il17ra:* Mm00434214_m1, *Il17b: Mm01258783_m1*, *Timp1: Mm01341361_m1*, *Tgfb1: Mm01178820_m1*, *Tgfb2: Mm00436955_m1*, *Tnfa: Mm00443258_m1*, *Mmp12: Mm00500554_m1*, *Ddr1: Mm01273496_m1*.

### Histologic evaluation

Two weeks after the treatment was employed, animals were sacrificed and mouse whole joints were fixed in 4% paraformaldehyde (PFA), decalcified for approximately 2 weeks in 10% EDTA. After decalcification, the samples were dehydrated and embedded in paraffin. The entire joints were sectioned at a thickness of 7 μm and stained for proteoglycans using Safranin-O and Fast Green staining (Applied Biosciences) according to the manufacturer’s protocol. Samples were imaged at × 20 magnification on an Axio Observer.Z1 (Zeiss) microscope with Axiocam 305 color imaging device. Köhler alignment was performed prior to imaging. Tiled images (2 × 2) were taken around each joint in the brightfield contrast method. The images were then rotated and cropped in Adobe Photoshop CS5 Extended Version 12.0 × 64 to align all joints.

### Immunofluorescence staining

Human CD4 expression was evaluated using immunofluorescence. FFPE joint 7 μm sections were deparaffinized and re-fixed using standard protocols. Heat-mediated antigen retrieval was performed in a citrate buffer pH 6 (PerkinElmer, AR600250ML) for 15 min at 95 °C. Endogenous peroxidases were blocked using 3% H_2_O_2_ for 15 min. Sections were blocked with a 10% BSA in TBS-T (Sigma, Thermo Scientific) buffer for 30 min and incubated overnight with CD4 primary antibodies (1:500, ab133616, Abcam) or IgG isotype control at the same concentration (ab172730, Abcam) in 10% BSA in TBS-T. MACH 3 Rabbit HRP Polymer Detection system was used according to the manufacturer’s protocol to detect the CD4 rabbit antibodies (BIOCARE MEDICAL, M3R531H). Tyramide signal amplification (TSA) visualization was performed using Opal 650 (PerkinElmer) according to the manufacturer’s protocol. Sections were rinsed with water and TBS-T, counterstained with DAPI for 5 min, mounted using DAKO mounting medium (Agilent), cover-slipped, and subsequently imaged. Isotype control and primary delete were used as controls using deidentified human surgical discard breast capsule samples, shown in Fig. S8. Samples were imaged at × 20 magnification on an Axio Observer.Z1 (Zeiss) microscope with an Axiocam 506 mono imaging device. Images were created by applying maximum intensity projections (MIP) in the Zen Blue software Version Zen 2.5 Pro.

### Hind limb weight-bearing assessment

Weight-bearing in mice was measured in the no surgery control and compared to ACLT groups receiving saline control or APS, CD3^+^, CD45^+^, or APS without cell treatment using an incapacitance tester (Columbus Instruments). The percentage of weight distributed on the ACLT limb was used as a marker for joint irritation in OA. The mice were positioned to stand on their hind paws in an angled box placed above the incapacitance tester. In this stance, each hind paw rested on a separate force plate where the force (*g*) applied by each limb was quantified. Three consecutive 3-s readings were recorded and averaged to obtain the mean score.

### Hind limb responsiveness

Mice were placed on a transparent caged hotplate at 55 °C. The latency period for hind limb response indicated by jumping or paw-lick was recorded as response time. Hind limb responses were measured prior to sacrificing the mice in all animal groups. Three responses were taken per mouse and averaged to obtain the individual mean response time.

### Statistics

Statistical analyses were performed using multiple unpaired *t*-tests where the statistical significance was determined without correction for multiple comparisons for the immune cell profile data and macrophage polarization data. For lymphocyte data, multiple unpaired *t*-tests with the Holm-Sidak method for multiple comparisons were used. For CD8^+^ intracellular data, unpaired *t*-test with two-tailed *p*-value was employed. Ordinary one-way ANOVA with Tukey’s multiple comparisons test was used for in vivo studies data. Unpaired *t*-test with two-tailed *p*-value was employed for the Luminex data. For all statistical tests, alpha = 0.05.

### Study approval

All procedures were approved by the Johns Hopkins University Animal Care and Use Committee and conducted in accordance with the protocol guidelines of Johns Hopkins University. Whole blood samples obtained from 4 human volunteers with valid consent (WIRB HBD-001 Study#: 1115097) approved by the WCG IRB committee (1019 39th Avenue SE Suite 120, Puyallup, WA 98374-2115). Subjects were deidentified. The breast capsule sample used as control samples for immunofluorescence staining were deidentified surgical discards from patients that were undergoing either breast implant exchange or replacement procedures.

## Supplementary Information

**Additional file 1: Fig. S1.** Gating strategy for identifying immune cells in APS samples. **Fig. S2.** **a**, Flow cytometric analysis of CD45^+^ immune cell subpopulations that are present in smaller concentrations in APS and WBCs (data are mean ±SD). **b**, Fold change for quantification of cell enrichment of immune subpopulations that are present in smaller concentrations in APS compared to WBCs, (whiskers represent min to max). Non-classical monocytes (NC Mono), Dendritic Cells (DCs), Eosinophils (Eos.), and Other HLA^+^. **c**, Heatmap of the differentially expressed genes after APS processing of sorted granulocytes compared to sorted granulocytes from WBCs. Bar plot of Log_2_(fold change) for significantly differentially expressed genes **d**, Sample correlation heatmap of all the Nanostring data ** e**, Multidimensional scaling (MDS) plot of all Nanostring data colored by immune cell type. Multiple unpaired T-test without correction for multiple comparisons, with set *P* value threshold, alpha=0.05 for (a). *p<0.05. **Fig. S3.** FACS Gating Strategy for Nanostring multiplex gene expression assay and RAG KO *in vivo* study. **Fig. S4.** **a**, Quantification of flow cytometry results for CD80^+^, double positive (Pos^++^), and double negative (Neg^--^) classical and non-classical monocyte concentrations in APS and WBCs. Individual donors denoted as circles for APS and triangles for WBCs. Data are mean with ± SD. **b**, APS to WBC cell count ratio of CD80^+^, CD163^+^, double positive (Pos^++^), and double negative (Neg^--^) populations in classical and non-classical monocytes, line at Y=5 **c**, STRING PPI network analysis for the differentially regulated genes from sorted monocyte Nanostring data comparing APS to WBCs using the STRING database version 11.0 and visualized with Cytoscape 3.9.0. Essential genes identified using the CytoNCA plugin are in green. PPI depicts the physical subnetwork where the gray edges indicate that the proteins are part of a physical complex. Active interaction sources were from textmining, experiments, databases, co-expression, neighborhood, gene fusion, and co-occurrence. The minimum required interaction score was 0.4. Disconnected nodes are not shown. **d**, PPI subnetwork of the most significant module with 19 nodes in orange and their first neighbors identified by MCODE, molecular complex detection algorithm. Blue nodes represent the first neighbors. Multiple unpaired T-test without correction for multiple comparisons, with set *P* value threshold, alpha=0.05 for (a). *p<0.05. **Fig. S5.** Lymphocyte intracellular staining gating strategy. **Table S1.** Antibodies used for flow cytometry analysis. **Fig. S6.** **a**, Luminex cytokine assay results for TGF-𝛃1, TGF-𝛃2, IGF-1, TNF𝛂, IL-1𝛃, and IL-1Ra in APS and blood (data mean ± SD). ND=non-detectible concentration. **b**, Luminex cytokine assay results. Unpaired t-test with two-tailed p-value, alpha=0.05 for (a-b), ***p<0.001, ****p<0.0001. **Fig. S7.** **a**, Quantification of mRNA expression for *Il1b, Il10, Ifng, Il4, Il17* in inguinal lymph node tissue (LN) (data are mean ± SEM). **b**, Quantification of mRNA expression for *Timp1, Tgfb1, Tgfb2, Il17ra, IL17b, Tnfa, Il1b, Mmp12*, and *Ddr1* in the articular joint (data are mean ± SEM). Ordinary one-way ANOVA with Tukey’s multiple comparison tests for (a-b). *p<0.05, **p<0.01, ***p<0.001. **Fig. S8.** **a**, Control samples for Immunofluorescence staining of human CD4^+^ cells (pink), that does not react with mouse and DAPI counterstain (blue) (20x magnification). Scale bar= 50 μm. Human breast capsule samples were used for isotype, positive, and primary delete controls.

## Data Availability

All relevant data in this manuscript are included in the published manuscript or supplementary materials.
