# Supplementary material for: Autologous Protein Solution processing alters lymphoid and myeloid cell populations and modulates gene expression dependent on cell type
Source: Arthritis Res Ther. 2022 Sep 12;24:221. doi: 10.1186/s13075-022-02875-x (PMC9465964; doi:10.1186/s13075-022-02875-x)
Supplement: Supplementary file 1 — Additional file 1: Fig. S1. Gating strategy for identifying immune cells in APS samples. Fig. S2. a, Flow cytometric analysis of CD45+ immune cell subpopulations that are present in smaller concentrations in APS and WBCs (data are mean ±SD). b, Fold change for quantification of cell enrichment of immune subpopulations that are present in smaller concentrations in APS compared to WBCs, (whiskers represent min to max). Non-classical monocytes (NC Mono), Dendritic Cells (DCs), Eosinophils (Eos.), and Other HLA+. c, Heatmap of the differentially expressed genes after APS processing of sorted granulocytes compared to sorted granulocytes from WBCs. Bar plot of Log2(fold change) for significantly differentially expressed genes d, Sample correlation heatmap of all the Nanostring data e, Multidimensional scaling (MDS) plot of all Nanostring data colored by immune cell type. Multiple unpaired T-test without correction for multiple comparisons, with set P value threshold, alpha=0.05 for (a). *p<0.05. Fig. S3. FACS Gating Strategy for Nanostring multiplex gene expression assay and RAG KO in vivo study. Fig. S4. a, Quantification of flow cytometry results for CD80+, double positive (Pos++), and double negative (Neg--) classical and non-classical monocyte concentrations in APS and WBCs. Individual donors denoted as circles for APS and triangles for WBCs. Data are mean with ± SD. b, APS to WBC cell count ratio of CD80+, CD163+, double positive (Pos++), and double negative (Neg--) populations in classical and non-classical monocytes, line at Y=5 c, STRING PPI network analysis for the differentially regulated genes from sorted monocyte Nanostring data comparing APS to WBCs using the STRING database version 11.0 and visualized with Cytoscape 3.9.0. Essential genes identified using the CytoNCA plugin are in green. PPI depicts the physical subnetwork where the gray edges indicate that the proteins are part of a physical complex. Active interaction sources were from textmining, experime [file 13075_2022_2875_MOESM1_ESM.docx]

Additional file 1

**Fig. S1.** Gating strategy for identifying immune cells in APS samples.


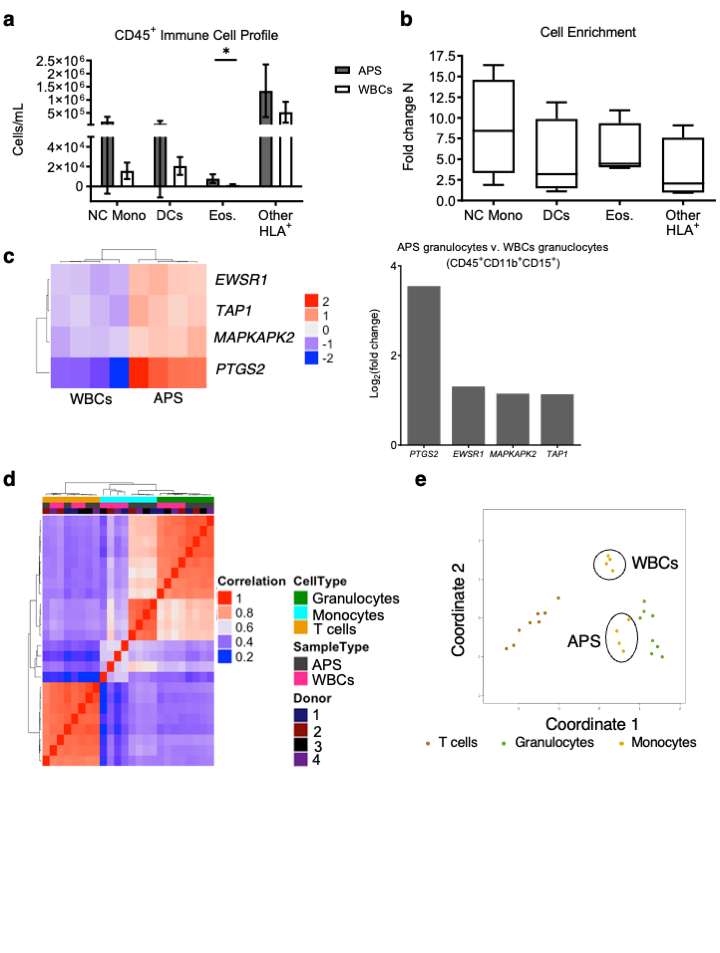


**Fig. S2. a,** Flow cytometric analysis of CD45^+^ immune cell subpopulations that are present in smaller concentrations in APS and WBCs (data are mean ±SD).  **b,** Fold change for quantification of cell enrichment of immune subpopulations that are present in smaller concentrations in APS compared to WBCs, (whiskers represent min to max). Non-classical monocytes (NC Mono), Dendritic Cells (DCs), Eosinophils (Eos.), and Other HLA^+^. **c,** Heatmap of the differentially expressed genes after APS processing of sorted granulocytes compared to sorted granulocytes from WBCs. Bar plot of Log_2_(fold change) for significantly differentially expressed genes **d,** Sample correlation heatmap of all the Nanostring data **e,** Multidimensional scaling (MDS) plot of all Nanostring data colored by immune cell type. Multiple unpaired T-test without correction for multiple comparisons, with set P value threshold, alpha=0.05 for (**a**). *p<0.05.


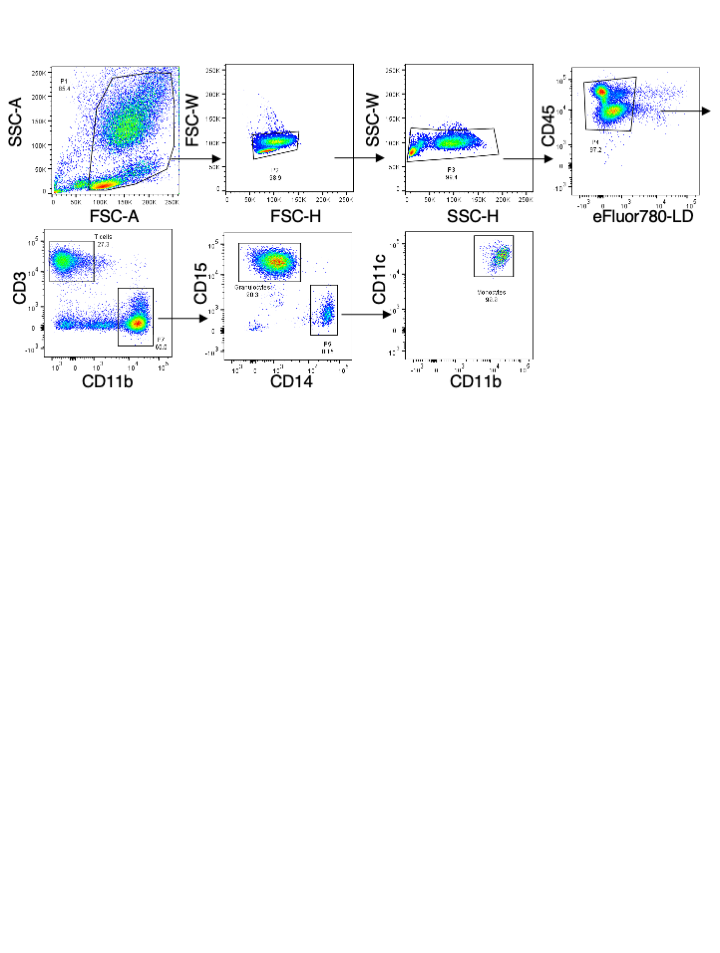


**Fig. S3.** FACS Gating Strategy for Nanostring multiplex gene expression assay and RAG KO *in vivo* study.


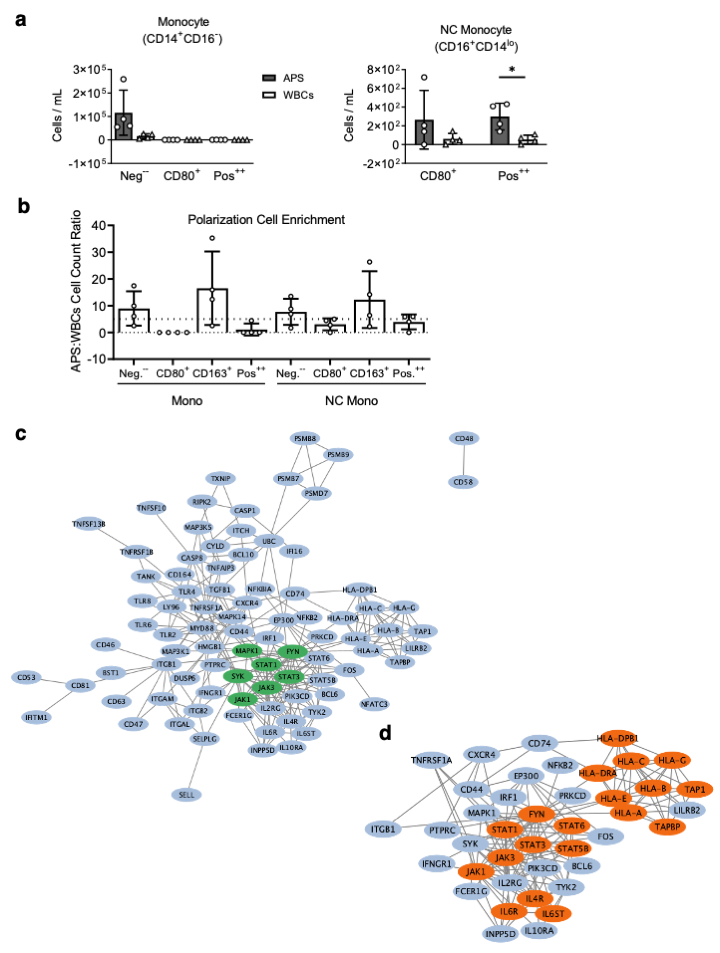


**Fig. S4. a,** Quantification of flow cytometry results for CD80^+^, double positive (Pos^++^), and double negative (Neg^--^) classical and non-classical monocyte concentrations in APS and WBCs. Individual donors denoted as circles for APS and triangles for WBCs. Data are mean with ± SD. **b,** APS to WBC cell count ratio of CD80^+^, CD163^+^, double positive (Pos^++^), and double negative (Neg^--^) populations in classical and non-classical monocytes, line at Y=5 **c,** STRING PPI network analysis for the differentially regulated genes from sorted monocyte Nanostring data comparing APS to WBCs using the STRING database version 11.0 and visualized with Cytoscape 3.9.0. Essential genes identified using the CytoNCA plugin are in green. PPI depicts the physical subnetwork where the gray edges indicate that the proteins are part of a physical complex. Active interaction sources were from textmining, experiments, databases, co-expression, neighborhood, gene fusion, and co-occurrence. The minimum required interaction score was 0.4. Disconnected nodes are not shown. **d,** PPI subnetwork of the most significant module with 19 nodes in orange and their first neighbors identified by MCODE, molecular complex detection algorithm. Blue nodes represent the first neighbors. Multiple unpaired T-test without correction for multiple comparisons, with set P value threshold, alpha=0.05 for (**a**). *p<0.05.


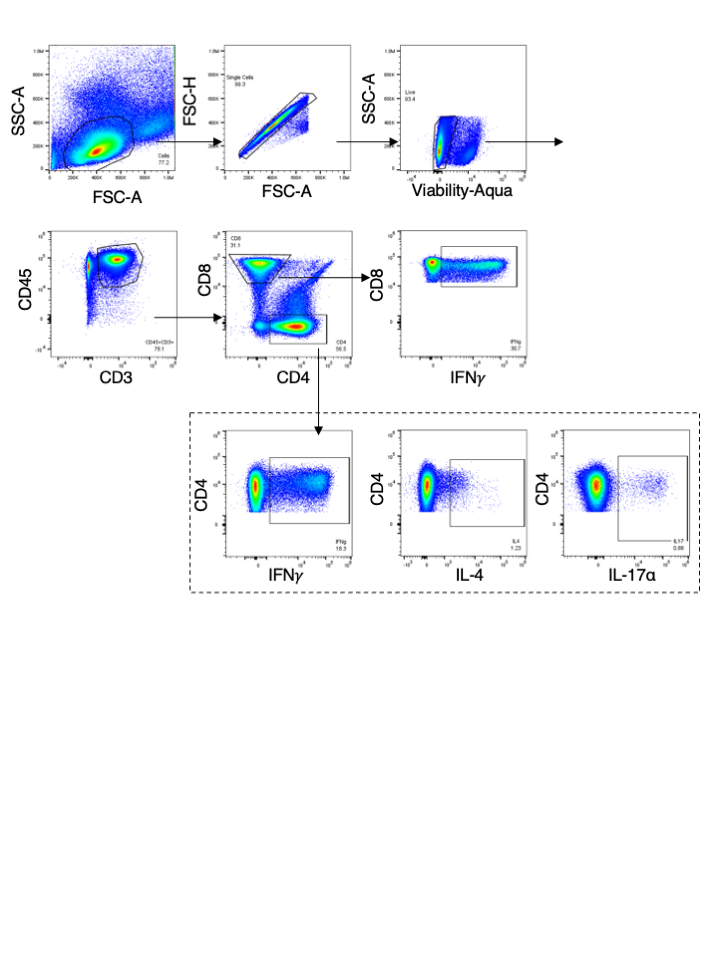


**Fig. S5.** Lymphocyte intracellular staining gating strategy.


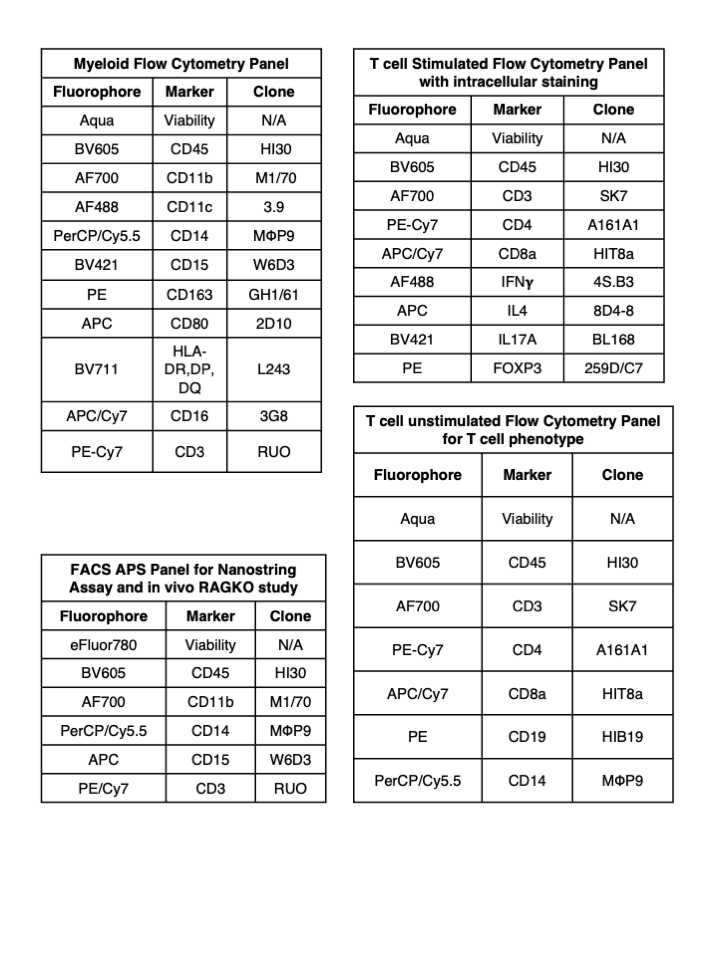


**Table S1.** Antibodies used for flow cytometry analysis.


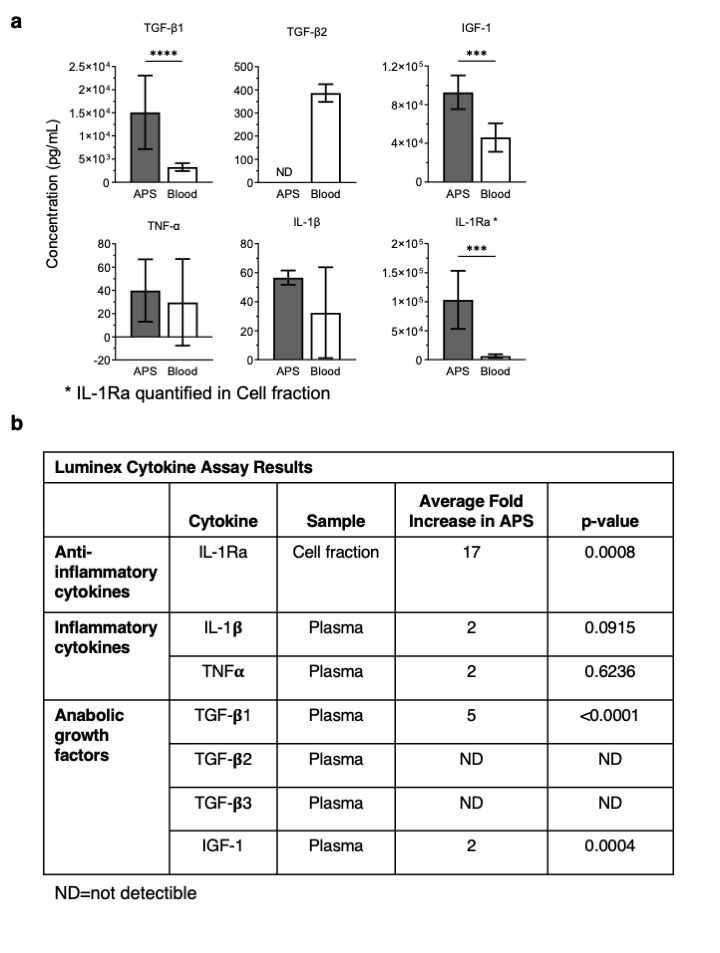


**Fig. S6. a,** Luminex cytokine assay results for TGF-𝛃1, TGF-𝛃2, IGF-1, TNF𝛂, IL-1𝛃, and IL-1Ra in APS and blood (data mean ± SD). ND=non-detectible concentration. **b,** Luminex cytokine assay results. Unpaired t-test with two-tailed p-value, alpha=0.05 for (**a-b**), ***p<0.001, ****p<0.0001.


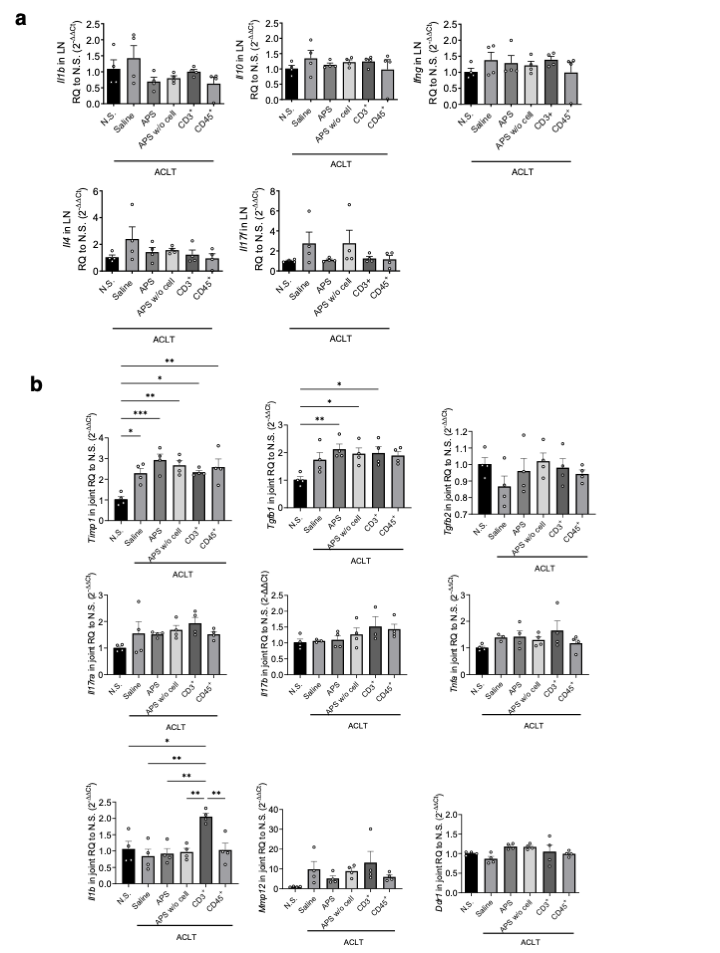


**Fig. S7.** **a,** Quantification of mRNA expression for *Il1b, Il10, Ifng, Il4, Il17* in inguinal lymph node tissue (LN) (data are mean ± SEM). **b,** Quantification of mRNA expression for *Timp1, Tgfb1, Tgfb2, Il17ra, IL17b, Tnfa, Il1b, Mmp12*, and *Ddr1* in the articular joint (data are mean ± SEM). Ordinary one-way ANOVA with Tukey’s multiple comparison tests for (**a-b**). *p<0.05, **p<0.01, ***p<0.001.


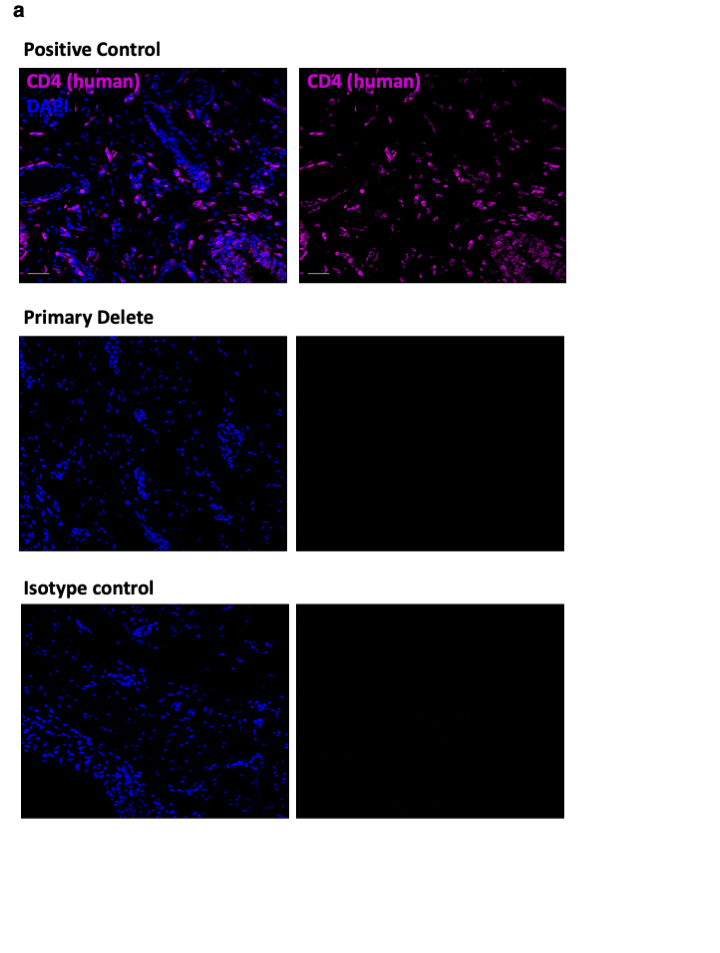


**Fig. S8.** **a,** Control samples for Immunofluorescence staining of human CD4^+^ cells (pink), that does not react with mouse and DAPI counterstain (blue) (20x magnification). Scale bar= 50 μm. Human breast capsule samples were used for isotype, positive, and primary delete controls.
